# Maternal physical activity before IVF/ICSI cycles improves clinical pregnancy rate and live birth rate: a systematic review and meta-analysis

**DOI:** 10.1186/s12958-018-0328-z

**Published:** 2018-02-07

**Authors:** Meng Rao, Zhengyan Zeng, Li Tang

**Affiliations:** 1 Department of reproduction and genetics, the First Affiliated Hospital of Kunming Medical University, No. 295 Xi Chang road, Kunming, 650032 China; 2 Department of Neurology, the First Affiliated Hospital of Kunming Medical University, Kunming, 650032 China

**Keywords:** Physical activity, ART, IVF/ICSI, Reproductive outcome

## Abstract

**Objective:**

This meta-analysis was aimed to evaluate the association between maternal physical activity before IVF/ICSI cycles and reproductive outcomes.

**Methods:**

We searched databases of PubMed, EMBASE and Web of Science electronic databases, and ongoing trials up to November 2017 to identify studies that focused on the relationship between maternal physical activity before IVF/ICSI cycles and reproductive outcomes, including implantation rate, clinical pregnancy rate, miscarriage rate and live birth rate. Odds ratio (OR) with 95% confidence intervals, were calculated to assess the results of each outcome.

**Results:**

Eight published studies encompassing 3683 infertile couples undergoing IVF/ICSI treatment were included into the analysis. There was an increasing, but not statistically significant, trend in implantation rate for physically active women when compared with physically inactive women (OR = 1.95, 95% CI 0.99–3.83, *I*^2^ = 77%). No significant difference was found in miscarriage rate between physically active women and physically inactive women (OR = 0.76, 95% CI 0.41–1.44, *I*^2^ = 49%). However, rates of clinical pregnancy and live births in physically active women were significantly higher than those in physically inactive women (OR = 1.96, 95% CI 1.40, 2.73, *I*^2^ = 42% and OR = 1.95, 95% CI 1.06–3.59, *I*^2^ = 82%, respectively). Subgroup analysis helped to confirm these results.

**Conclusions:**

Female physical activity before IVF/ICSI cycles was associated with increased rates of clinical pregnancy and live births, whereas only a small but not statistically significant increase was found in implantation rate, and no effect was shown on miscarriage rate.

## Background

The prevalence of infertility is estimated to be 12–15% in couples of childbearing age [1, 2]. Even though the development of assisted reproductive technology (ART) has helped many couples achieve pregnancies, the rates of pregnancy and live births among all ART-treated couples is still not satisfactory. How to improve assisted reproductive outcome has become a critical topic for both infertile couples and clinicians.

Physical activity is generally considered to be a health promoting behaviour as it is associated with reduced risks of cardiovascular disease, diabetes and cancers [3, 4]. A number of epidemiological studies have focused on the effects of physical activity on fertility, but no consensus has been reached until now [5–7]. Most of the investigators reported that moderate physical activity benefits female fertility [5–7], whereas high intensity and frequency of physical activity increase subfertility and infertility, mainly ovulatory infertility [6], and increasing vigorous physical activity is associated with delayed time to spontaneous pregnancy [7]. Similar conclusions were also reached in athletes, who have been shown to have a higher prevalence of reproductive dysfunction compared with non-athletes [8, 9].

Physical activity during pregnancy has been shown to improve reproductive outcome [10, 11]. However, no consensus has been reached regarding the effect of physical activity before ART on pregnancy outcome [12–14]. A population-based cohort study [12] showed that women engaged in physical activity for ≥4 h/week had a 40% decreased likelihood of live birth in in vitro fertilization (IVF) cycles compared with women not regularly engaged in physical activity. This conclusion was supported by some other investigations focusing on IVF or intracytoplasmic sperm injection (ICSI) cycles [13, 15, 16]. Whereas a randomized controlled trial (RCT) [17] showed no differences in pregnancies or live births between the physical activity intervention and control groups. Gaskins et al. [14] also found that time spent in moderate to vigorous physical activities and total metabolic equivalent task hours before IVF were not associated with probability of implantation, clinical pregnancy or live birth.

Therefore, in this systemic review and meta-analysis, we aimed to evaluate the association between maternal physical activity before IVF/ICSI cycles and reproductive outcomes, to provide a comprehensive analysis of the current data and a context for how to counsel infertile couples and physicians trying to improve the success rate of assisted reproductive treatment.

## Methods

### Literature search

A systematic literature review was performed to identify all published studies on PubMed, EMBASE and Web of Science electronic databases, and Clinicaltrails.gov up to November 2017. The search was limited to human studies published in English. The following terms were used to search the databases: (“physical activity OR physical active OR physical non-active OR exercise”) AND (“assisted reproductive OR IVF OR ICSI”). The reference lists of the relevant publications were also manually searched for related studies. Two researchers independently completed the literature search and identified the eligible studies. Conflicting decisions were resolved through consensus with a third researcher. The studies were included if they satisfied the following criteria: 1) infertile couples treated with ART, 2) pregnancy outcomes were compared between physical active and non-active women, or women with different levels of physical activity, and 3) pregnancy outcomes included implantation rate, clinical pregnancy rate, miscarriage rate or live birth rate. The implantation rate should be calculated as the ratio of the number of gestational sacs/number of transferred embryos; the clinical pregnancy should be diagnosed by ultrasonography 4 weeks after embryo transfer; the miscarriage rate was defined as the number of miscarriages in the first 20 weeks of gestation per clinical pregnancy; and the live birth rate was defined as the number of deliveries that resulted in at least one live-born baby per initiated cycle. The studies were excluded if 1) the subjects were athletes; 2) they were published as an abstract, letter to editor, case report or review; and 3) they failed to provided enough data for data analysis.

### Data extraction

Two reviewers independently extracted the data from the included articles according to the following information: first author, year of publication, country, number of patients, age, body mass index (BMI), physical activity intensity, treatment and pregnancy outcomes. We needed the data to be expressed as the odds ratio (OR) and 95% confidence interval (CI). If not, the data were extracted and then transferred to this form according to specific statistical methods. In addition, data from different subgroups were extracted for possible synthesis if necessary. The corresponding author was contacted for more information if the data presented in the article was inadequate for analysis.

### Quality assessment of included studies

A total of 8 studies were eligible and enrolled into the meta-analysis, 7 of which were cohort studies, so the Newcastle-Ottawa Quality Assessment Scale (NOS) was used for the quality assessment [18]. The NOS evaluates a high quality study from the three aspects: selection of participants, comparability of study groups, and the ascertainment of outcomes of interest. A score of 6–9 is considered to suggest a high quality and low risks of bias. Discrepancies were resolved through consensus. For another randomised controlled trial (RCT) published by Moran et al. [17], the Cochrane risk of bias tool was applied to evaluate the quality based on randomization, blinding of outcome assessment, completeness of outcome assessment, selective reporting, and other bias [19]. Each domain was categorized as low, high, or unclear.

### Quality of evidence

The quality of the evidence for all outcomes were assessed using the criteria of the Grading of Recommendations Assessment, Development and Evaluation (GRADE) system (study limitations, consistency of effect, imprecision, indirectness, and publication bias), which specifies four levels of evidence: high, moderate, low, and very low quality evidence [20, 21]. The quality of evidence could be downgraded by one or two levels if serious or very serious deficiencies in these criteria were considered.

### Statistical analysis

All analyses were performed with Review Manager version 5.2 software (The Cochrane Collaboration). A standard meta-analytic method was used to compare the studies included in this study, and the odds ratio (OR) with its 95% CI was applied to express the combined result. A random-effects model was used. The degree of heterogeneity was also measured by the *I*^2^ statistic, where *I*^2^ > 50% was regarded as an indicator of significant heterogeneity [22]. Evaluation of inter-study variance was performed by calculating Tau^2^, which represents the estimated standard deviation of underlying effects across studies. Subgroup analysis was also conducted to further analyze the effect of physical activity on pregnancy outcome based on the following aspects: controlling for important potential confounders, principally age and BMI (Yes or No) and whether the intensity of exercise in physically active women was > 2.5 h/week in each study (Yes or No/Not clear). The level of statistical significance was set at *P* < 0.05.

## Results

### Literature search

**Fig. 1:**
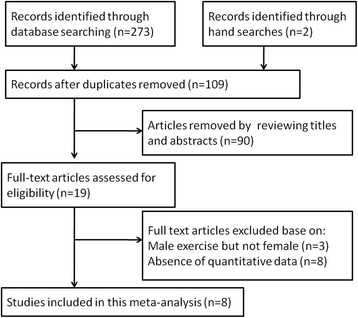
Flow chart for selection of eligible studies

The literature search resulted in 273 articles for review, including 2 additional studies identified by manual search. After removing duplicate studies and reviewing titles and abstracts, 19 full-text articles were screened and assessed for eligibility. Thereafter, another 11 articles were excluded because these studies either focused on male but not female physical activity (*n* = 3), or they did not provide data on implantation rate, pregnancy rate, miscarriage rate or live birth rate (*n* = 8). Finally, 8 full-text studies comprising 3683 infertile couples were included in this meta-analysis [12–17, 23, 24]. Figure 1 shows the flow diagram of the selection process. Morris et al. [12] collected the data on physical activity by using a simple questionnaire described in the text. Ferreira et al. [23] used a validated lifestyle questionnaire reported elsewhere [25]. Both Kucuk et al. [16] and Ramezanzadeh et al. [24] used the original International Physical Activity Questionnaires in their studies (http://uacc.arizona.edu/sites/default/files/ipaq_english_telephone_short.pdf). The physical activity in the Moran et al. [17] study was a home-based physical conditioning and walking program as described elsewhere [26]. Evenson et al. [15] used a modified Kaiser Physical Activity Survey [27] that was described in detail in another study [28]. The questionnaire used in the Palomba et al. [13] study was formulated on the basis of the well-validated Global Physical Activity Questionnaire (www.who.int/chp/steps/GPAQ%20Instrument%20and%20Analysis%20 Guide%20v2.pdf). In the Gaskins et al. [14] study, another validated questionnaire was used for data collection [29]. The characteristics of these studies are summarized in Table 1.

**Table 1:** Summary of included studies

Author/year	No. of infertile couples	Treatment	Study type	Contrast	Evaluation of physical activity	Controlling for confounders	Pregnancy outcomes
Morris/2006	2232	IVF	Prospective	Regular exercise > 1 h/week versus no regular exercise	Simple questionnaires	Age, BMI	Miscarriage rate; Live birth rate
Ferreira/2010	436	ICSI	Prospective	Regular exercise at least 1 h for 3 times/week versus no regular exercise	Validated lifestyle questionnaire	Age	Miscarriage rate; Clinical pregnancy rate
Kucuk/2010	131	ICSI	Prospective	Moderate physical activity versus low physical activity	IPAQ ^a^	Age, BMI	Implantation rate; Clinical pregnancy rate; Miscarriage rate; Live birth rate
Moran/2011	38	IVF	Prospective	Exercise and diet intervention versus control	Patients participated in the Comprehensive Lifestyle Intervention Program (CLIP)	Age, BMI	Clinical pregnancy rate; Miscarriage rate; Live birth rate
Ramezanzadeh /2012	236	IVF/ICSI	Prospective	More than 3 h/week moderate-intensity exercise or 5 h/week low-intensity exercise versus not	IPAQ ^a^	None	Implantation rate; Clinical pregnancy rate
Evenson/2014	121	IVF	Prospective	Total activity index ≥10.6 versus < 10.6	Modified Kaiser Physical Activity Survey	Age, BMI	Clinical pregnancy rate; Live birth rate
Palomba/2016	216	IVF/ICSI	Prospective	Regular physical activity versus no regular physical activity	Self-administered semiquantitative general health questionnaires formulated on the basis of Global Physical Activity Questionnaire	Age, BMI	Implantation rate; Clinical pregnancy rate; Miscarriage rate; Live birth rate
Gaskins/2016	273	IVF	Prospective	Total physical activity> 2.5 h/week versus ≤2.5 h/week	A validated questionnaire	None	Clinical pregnancy rate; Miscarriage rate; Live birth rate

^a^*IPAQ* International Physical Activity Questionnaires

### Quality assessment

As shown in Table 2, the observational studies had a Newcastle-Ottawa Scale score ranging from 7 to 8, suggesting a low risk of bias. The category most often missed by studies was comparability because only age and/or BMI were adjusted for confounding in all included studies, many other factors like duration of infertility, male factors etc. were also critical for the outcomes of ART. The RCT had low risk of bias in the domains of random sequence generation, blinding of outcome assessment, incomplete outcome data and selective reporting, and had high risk of bias in allocation concealment. Whereas there was unclear risk of bias in the aspect of blinding of participants and personnel.

**Table 2:** Quality assessment of included studies

Author/year	Selection	Exposure	Comparability	Total score
Morris/2006	3	3	1	7
Ferreira/2010	4	3	1	8
Kucuk/2010	4	3	1	8
Ramezanzadeh/2012	4	3	0	7
Evenson/2014	4	3	1	8
Palomba/2016	4	3	1	8
Gaskins/2016	4	3	0	7

Newcastle-Ottawa Quality Assessment Scale (NOS) was used for the quality assessment

### Implantation rate

**Fig. 2:**
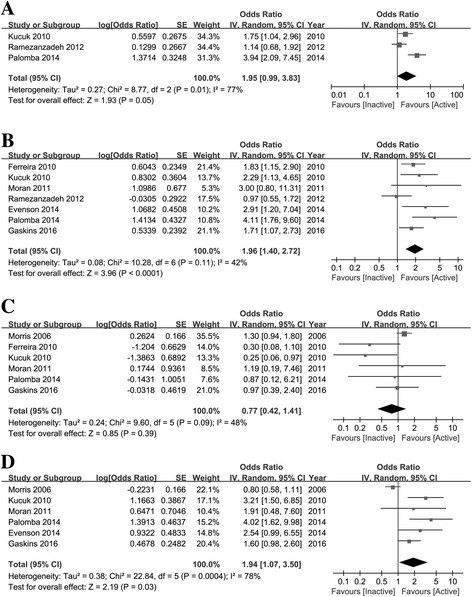
Forest plot (random-effects model) of physical activity before IVF/ICSI cycles and pregnancy outcome following IVF/ICSI cycles. **a** implantation rate; (**b**) clinical pregnancy rate; (**c**) miscarriage rate and (**d**) live birth rate

Only three of the eight included studies reported the implantation rate [13, 16, 24], of which two showed improvement of the implantation rate, and the other presented no obvious association. As a group, the studies in this meta-analysis did not show a significant difference in implantation rate for women performing regular physical activity; however, there was a trend toward the association of physical activity before IVF/ICSI cycles with implantation rate (OR = 1.95, 95% CI 0.99–3.83, *n* = 583, *I*^2^ = 77%, low quality evidence), even though heterogeneity existed (Fig. 2).

### Clinical pregnancy rate

Seven of the included studies presented data on clinical pregnancy rate [13–17, 23, 24]. Five of the studies showed a significant increase in clinical pregnancy rate among women who undertook regular physical activity, and the remaining articles failed to find any association. Meta-analysis of all 7 of these studies showed an overall increase in clinical pregnancy rate among those undertaking regular exercise compared with no regular exercise (OR = 1.96, 95% CI 1.40–2.73, *n* = 1451, *I*^2^ = 42%, moderate quality evidence), with non-significant heterogeneity (Fig. 2). In 4 of the 7 studies, age and BMI were controlled between the physically active and inactive women. When combining these studies, subgroup meta-analysis showed an increase in the clinical pregnancy rate among physically active women when compared with physically inactive women (OR = 2.92, 95% CI 1.89–4.52). The other three studies, which were not well controlled for age and BMI, also showed a significant increase in clinical pregnancy rate when the subjects undertook regular physical activity (OR = 1.50, 95% CI 1.04–2.15). In three of the seven studies, physically active women exercised for more than 2.5 h/week, and when we combined these studies, the results showed a 1.49-fold increase in clinical pregnancy rate for physically active women in comparison with control subjects (95% CI 1.04–2.15). In the other four studies, the authors did not clearly present the intensity of physical activity, or the exercise intensity in the physically active women was not more than 2.5 h/week. Nevertheless, the combination of these studies still showed an increase in the clinical pregnancy rate for physically active women (OR = 2.92, 95% CI 1.89–4.52), as shown in Table 3.

**Table 3:** Subgroup analysis for the effect of maternal physical activity on pregnancy outcome following IVF/ICSI

Subgroup	No. of studies	OR (95% CI)	Tau^2^	*I* ^2^	P_*subgroup*_
Age, BMI controlling					
Clinical pregnancy rate					
Yes	4	2.92 (1.89–4.52)	0	0	
No	3	1.50 (1.04–2.15)	0.04	38	0.02
Miscarriage rate					
Yes	4	0.85 (0.37–1.92)	0.32	46	
No	2	0.59 (0.19–1.84)	0.36	52	0.62
Live birth rate					
Yes	5	2.10 (0.90–4.86)	0.72	85	
No	1	1.60 (0.98–2.60)	N/A	N/A	0.58
Exercise > 2.5 h/week in physical active women					
Clinical pregnancy rate					
Yes	4	1.56 (1.11–2.21)	0.03	27	
NO/ Not clear^a^	3	2.91 (1.83–4.62)	0	0	0.03
Miscarriage rate					
Yes	3	0.71 (0.32–1.55)	0.09	16	
NO/ Not clear^a^	3	0.70 (0.19–2.56)	0.77	64	0.99
Live birth rate					
Yes	2	1.65 (1.07–2.56)	0	0	
NO/ Not clear^a^	4	2.16 (0.84–5.54)	0.79	88	0.62

The subgroup analysis was not conducted on implantation rate since there were only 3 studies reported this data

*OR* odds ratio, *CI* confidence interval, *N/A* not applicable

^a^Intensity of physical activity was not > 2.5 h/week in physical active women in these studies, or the data was not clearly described

### Miscarriage rate

Six articles included data on miscarriage rate, and all showed no significant difference in miscarriage rate between physically active and inactive women [12–14, 16, 17, 23]. The meta-analysis showed no significant association of physical activity with miscarriage rate (OR = 0.76, 95% CI 0.41–1.44, *n* = 3326, *I*^2^ = 49%, low quality evidence), with an acceptable heterogeneity (Fig. 2). In subgroup analysis, the combined results were very similar between the studies controlled for age and BMI and those in which age and BMI were not well controlled (OR = 0.85, 95% CI 0.37–1.92 and OR = 0.59, 95% CI 0.19–1.84, respectively). Combined results for age and BMI were also very consistent when the studies were stratified by exercise intensity in the physically active women (OR = 0.60, 95% CI 0.19–1.85 and OR = 0.83, 95% CI 0.34–2.06, respectively), as shown in Table 3.

### Live birth rate

Six studies reported data on live birth rate [12–17], among which 2 studies showed a significant increase in live birth rate for women who exercised regularly when compared with those who did not. Two other studies presented an increasing, but not statistically significant, trend for live birth rate. Additionally, another population-based study indicated a decreasing trend in live birth rate for physical active women even though the decrease was not statistically significant. The meta-analysis showed a positive association between physical activity and live birth rate (OR = 1.95, 95% CI 1.06–3.59, *n* = 3011, *I*^2^ = 82%, low quality evidence), but heterogeneity existed (Fig. 2). In the subgroup analysis, combination of the results of the 5 studies controlled for age and BMI showed no obvious effect of physical activity on the live birth rate (OR = 2.10, 95% CI 0.90–4.86, *I*^2^ = 85%), whereas the pooled results changed significantly when the study by Morris et al. [12] was removed (OR = 3.03, 95% CI 1.90–4.84, *I*^2^ = 0). The combined results showed an increase in live birth rate among women undertaking regular exercise (OR = 1.63, 95% CI 1.03–2.58, *I*^2^ = 0) for those studies in which exercise intensity was > 2.5 h/week. Nevertheless, the live birth rate was not significantly affected in the studies in which exercise intensity was not > 2.5 h/week or not clearly described (OR = 2.16, 95% CI 0.84–5.54, *I*^2^ = 88%), as shown in Table 3.

## Discussion

To our knowledge, this is the first meta-analysis to evaluate the relationship between female physical activity before IVF/ICSI cycles and ART outcomes. We found that physical activity before IVF/ICSI cycles is associated with better assisted reproductive outcomes, mainly based on the increase in the rates of clinical pregnancy and live birth, and also a small but not statistically significant increase in the implantation rate, whereas the miscarriage rate was not associated with physical activity in women before ART cycles. These results suggested that exercise before IVF/ICSI cycles may clearly help physically inactive women to improve their chance of a successful pregnancy.

The effect of exercise on fertility and IVF outcomes has been a subject of considerable dispute. A recent population-based cohort study indicated that moderate exercise is associated with better clinical outcomes, regardless of BMI [7]. In 2008, the (United States Department of Health and Human Services (USDHHS) released in the “Physical Activity Guidelines for Americans” which recommended at least 150 min of moderate-intensity physical activity per week for pregnant women without obstetric/medical complications [30]. However, until now, no population-based RCT was conducted to show whether moderate physical activity prior to IVF/ICSI was beneficial to ART outcomes. The only RCT enrolled 38 overweight/obese women who received lifestyle intervention (exercise and weight-loss diet) or standard treatment for 5–9 weeks before oocyte pick-up. The results showed no significant improvement of clinical pregnancy and live birth in intervention patients compared with control. However, the small sample size in that study may be insufficient to draw a solid conclusion. In this meta-analysis, we provided a comprehensive analysis of the current data and found a 1.96-fold and 1.94-fold increase of clinical pregnancy rate and live birth rate, respectively, in physical active women compared with physical inactive women. Whereas no significant differences were found in implantation rate and miscarriage rate. According to the GRADE criteria, the combined results were assessed with moderate to low quality evidence, since most included studies were prospective cohort studies, and cofounders like dietary pattern, duration of physical activity as well as male factors in patients would affect clinical outcomes. Additionally, heterogeneity among studies existed in every clinical outcome. Considering that the following two aspects may affect the combined results: 1) age and BMI were critical confounders affecting IVF/ICSI outcomes [27–29], but were not controlled in some of included studies; 2) Intensity of physical activity was not consistent among included studies, we performed sub-group analysis to further evaluate the relationship between physical activity before IVF/ICSI cycles and clinical outcomes. As a result, we found very similar pooled effects in comparison with the overall results, suggesting that the effect of physical activity on pregnancy outcome was independent of age and weight loss. Additionally, in most of the included studies, patients reduced their physical activity when pregnancy was achieved. This may help to exclude the effect of physical activity during pregnancy on assisted reproductive outcomes, and this has been shown in several studies [31–33].

The mechanisms by which physical activity prior to IVF/ICSI cycles improves pregnancy outcome may be very complex, and no molecular pathway has been identified. The most relevant determinants of regular physical activity on reproductive outcome seem to be mainly related to its effect on the clinical pregnancy rate. This may be due to the following reasons: First, physical activity performed to improve health status may promote changes in energy balance, which, in turn, is tightly correlated with the reproductive system [34]. Second, physical activity may improve the assisted reproductive outcome through insulin sensitization, restore ovarian function [35] and sensitize the ovary to clomiphene citrate during simple ovulation induction [36]. Many infertile women are characterized by obesity, which is positively associated with insulin resistance, for instance, in polycystic ovary syndrome [37, 38]. Regular physical activity is also known to be an effective therapeutic intervention to improve glucose homeostasis and insulin sensitivity [39]. Endometrial insulin resistance should also be noted because studies have proved that a reduction in insulin resistance at the endometrial level induced by insulin-sensitizing agents leads to changes in the expression of glucose transporter endometrial protein [40] and is associated with a declining risk for miscarriage and implantation failure in IVF cycles during clinical observation [41]. Additionally, regular physical activity can help relieve stress and anxiety, which have been shown to be important risk factors affecting the assisted reproductive outcome [33, 42].

The strength of this review is that we focused on the effect of a very common lifestyle choice on assisted reproductive outcome, and the conclusion is very critical when providing consultation to infertile couples. All included studies established a good contrast between no regular physical activity and regular physical activity, and the intensity of physical activity was within a normal range. This excluded the effect of high-intensity physical activity (e.g., that performed by athletes) on pregnancy outcome. This study also has several limitations. First, the number of included studies was limited. The information on physical activity was based on the memories of the infertile couples. However, all physical activity information of the participants was recorded before the IVF/ICSI cycles, and they did not know whether they would become pregnant, which suggests the truthfulness of the results. Second, the questionnaires in the included studies differed from each other and bias cannot be excluded, even though all studies established a reliable contrast regarding different levels of physical activity. Third, we could not eliminate the effects from other confounders, such as etiology of infertility, dietary habits and psychological factors, which may also affect assisted reproductive outcomes.

To provide better evidence regarding the relationship between physical activity before ART and clinical outcomes, carefully controlled and sufficiently powered intervention studies are needed, frequency, intensity and duration of physical activity and potential confounders like dietary pattern, male factor etc. should be considered.

## Conclusion

From this meta-analysis, we conclude that physical activity before IVF/ICSI cycles is associated with increased rates of clinical pregnancy and live births, whereas only a small but not statistically significant increase was found in the implantation rate and no effect was shown on the miscarriage rate following IVF/ICSI cycles. It is necessary to determine the status of physical activity in women when they come to the clinic for infertility treatment, and suggesting to physically inactive women that they do more exercise is expected to improve the pregnancy outcome. However, the conclusions reached from this meta-analysis are based primarily on observational studies, and population-based randomized clinical trials will be needed to confirm the results.

## Abbreviations

ART: Assisted reproductive technology
BMI: Body mass index
ICSI: Intracytoplasmic sperm injection
IVF: In vitro fertilization
OR: Odds ratio
RCT: Randomized controlled trial
